# Burden of Diarrhea in the Eastern Mediterranean Region, 1990–2013: Findings from the Global Burden of Disease Study 2013

**DOI:** 10.4269/ajtmh.16-0339

**Published:** 2016-12-07

**Authors:** Ibrahim Khalil, Danny V. Colombara, Mohammad Hossein Forouzanfar, Christopher Troeger, Farah Daoud, Maziar Moradi-Lakeh, Charbel El Bcheraoui, Puja C. Rao, Ashkan Afshin, Raghid Charara, Kalkidan Hassen Abate, Mohammed Magdy Abd El Razek, Foad Abd-Allah, Remon Abu-Elyazeed, Aliasghar Ahmad Kiadaliri, Ali Shafqat Akanda, Nadia Akseer, Khurshid Alam, Deena Alasfoor, Raghib Ali, Mohammad A. AlMazroa, Mahmoud A. Alomari, Rajaa Mohammad Salem Al-Raddadi, Ubai Alsharif, Shirina Alsowaidi, Khalid A. Altirkawi, Nelson Alvis-Guzman, Walid Ammar, Carl Abelardo T. Antonio, Hamid Asayesh, Rana Jawad Asghar, Suleman Atique, Ashish Awasthi, Umar Bacha, Alaa Badawi, Aleksandra Barac, Neeraj Bedi, Tolesa Bekele, Isabela M. Bensenor, Balem Demtsu Betsu, Zulfiqar Bhutta, Aref A. Bin Abdulhak, Zahid A. Butt, Hadi Danawi, Manisha Dubey, Aman Yesuf Endries, Imad D. A. Faghmous, Talha Farid, Maryam S. Farvid, Farshad Farzadfar, Seyed-Mohammad Fereshtehnejad, Florian Fischer, Joseph Robert Anderson Fitchett, Katherine B. Gibney, Ibrahim Abdelmageem Mohamed Ginawi, Melkamu Dedefo Gishu, Harish Chander Gugnani, Rahul Gupta, Gessessew Bugssa Hailu, Randah Ribhi Hamadeh, Samer Hamidi, Hilda L. Harb, Mohammad T. Hedayati, Mohamed Hsairi, Abdullatif Husseini, Nader Jahanmehr, Mehdi Javanbakht, Tariku Jibat, Jost B. Jonas, Amir Kasaeian, Yousef Saleh Khader, Abdur Rahman Khan, Ejaz Ahmad Khan, Gulfaraz Khan, Tawfik Ahmed Muthafer Khoja, Yohannes Kinfu, Niranjan Kissoon, Ai Koyanagi, Aparna Lal, Asma Abdul Abdul Latif, Raimundas Lunevicius, Hassan Magdy Abd El Razek, Azeem Majeed, Reza Malekzadeh, Alem Mehari, Alemayehu B. Mekonnen, Yohannes Adama Melaku, Ziad A. Memish, Walter Mendoza, Awoke Misganaw, Layla Abdalla Ibrahim Mohamed, Jean B. Nachega, Quyen Le Nguyen, Muhammad Imran Nisar, Emmanuel Kwame Peprah, James A. Platts-Mills, Farshad Pourmalek, Mostafa Qorbani, Anwar Rafay, Vafa Rahimi-Movaghar, Sajjad Ur Rahman, Rajesh Kumar Rai, Saleem M. Rana, Chhabi L. Ranabhat, Sowmya R. Rao, Amany H. Refaat, Mark Riddle, Gholamreza Roshandel, George Mugambage Ruhago, Muhammad Muhammad Saleh, Juan R. Sanabria, Monika Sawhney, Sadaf G. Sepanlou, Tesfaye Setegn, Karen Sliwa, Chandrashekhar T. Sreeramareddy, Bryan L. Sykes, Mohammad Tavakkoli, Bemnet Amare Tedla, Abdullah S. Terkawi, Kingsley Ukwaja, Olalekan A. Uthman, Ronny Westerman, Mamo Wubshet, Muluken A. Yenesew, Naohiro Yonemoto, Mustafa Z. Younis, Zoubida Zaidi, Maysaa El Sayed Zaki, Abdullah A. Al Rabeeah, Haidong Wang, Mohsen Naghavi, Theo Vos, Alan D. Lopez, Christopher J. L. Murray, Ali H. Mokdad

**Affiliations:** 1 Institute for Health Metrics and Evaluation, University of Washington, Seattle, Washington; 2 Department of Community Medicine, Gastrointestinal and Liver Disease Research Center, Iran University of Medical Sciences, Tehran, Iran; 3 Jimma University, Jimma, Ethiopia; 4 Aswan Faculty of Medicine, Aswan, Egypt; 5 Department of Neurology, Cairo University, Cairo, Egypt; 6 GlaxoSmithKline, Philadelphia, Pennsylvania; 7 Clinical Epidemiology Unit, Orthopedics, Department of Clinical Sciences Lund, Lund University, Lund, Sweden; 8 Health Services Management Research Center, Institute for Futures Studies in Health, Kerman University of Medical Sciences, Kerman, Iran; 9 University of Rhode Island, Kingston, Rhode Island; 10 The Hospital for Sick Children, Toronto, Canada; 11 University of Toronto, Toronto, Canada; 12 Murdoch Children's Research Institute, Melbourne, Australia; 13 University of Melbourne, Melbourne, Australia; 14 University of Sydney, Sydney, Australia; 15 Ministry of Health, Al Khuwair, Oman; 16 University of Oxford, Oxford, United Kingdom; 17 Kingdom of Saudi Arabia Ministry of Health, Riyadh, Saudi Arabia; 18 Division of Physical Therapy, Department of Rehabilitation Sciences, Jordan University of Science and Technology, Irbid, Jordan; 19 Ministry of Health, Jeddah, Saudi Arabia; 20 Charité Universitätsmedizin, Berlin, Germany; 21 Department of Internal Medicine, College of Medicine and Health Sciences, United Arab Emirates University, Al-Ain, United Arab Emirates; 22 King Saud University, Riyadh, Saudi Arabia; 23 Universidad de Cartagena, Cartagena de Indias, Colombia; 24 Ministry of Public Health, Beirut, Lebanon; 25 Department of Health Policy and Administration, College of Public Health, University of the Philippines, Manila, Philippines; 26 Department of Medical Emergency, School of Paramedic, Qom University of Medical Sciences, Qom, Iran; 27 South Asian Public Health Forum, Islamabad, Pakistan; 28 Graduate Institute of Biomedical Informatics, Taipei Medical University, Taipei, Taiwan; 29 Sanjay Gandhi Postgraduate Institute of Medical Sciences, Lucknow, India; 30 School of Health Sciences, University of Management and Technology, Lahore, Pakistan; 31 Public Health Agency of Canada, Toronto, Canada; 32 Faculty of Medicine, University of Belgrade, Belgrade, Serbia; 33 College of Public Health and Tropical Medicine, Jazan, Saudi Arabia; 34 Madawalabu University, Bale Goba, Ethiopia; 35 University of São Paulo, São Paulo, Brazil; 36 Mekelle University, Mekelle, Ethiopia; 37 Medical Center, Aga Khan University, Karachi, Pakistan; 38 University of Iowa Hospitals and Clinics, Iowa City, Iowa; 39 Al Shifa Trust Eye Hospital, Rawalpindi, Pakistan; 40 Walden University, Minneapolis, Minnesota; 41 International Institute for Population Sciences, Mumbai, India; 42 Arba Minch University, Arba Minch, Ethiopia; 43 London School of Hygiene and Tropical Medicine, London, United Kingdom; 44 University of Louisville, Louisville, Kentucky; 45 Harvard T.H. Chan School of Public Health, Harvard University, Boston, Massachusetts; 46 Institute for Health Policy, Boston, Massachusetts; 47 Non-Communicable Diseases Research Center, Endocrinology and Metabolism Research Institute, Tehran University of Medical Sciences, Tehran, Iran; 48 Department of Neurobiology, Care Sciences and Society, Karolinska Institute, Stockholm, Sweden; 49 Bielefeld University, Bielefeld, Germany; 50 Harvard University, Boston, Massachusetts; 51 Department of Epidemiology and Preventive Medicine, Monash University, Melbourne, Australia; 52 Melbourne Health, Parkville, Australia; 53 College of Medicine, University of Hail, Hail, Saudi Arabia; 54 Haramaya University, Dire Dawa, Ethiopia; 55 Kersa Health and Demographic Surveillance System, Harar, Ethiopia; 56 Department of Microbiology, Saint James School of Medicine, Anguilla, British West Indies; 57 Department of Epidemiology and Biostatistics, Saint James School of Medicine, Anguilla, British West Indies; 58 West Virginia Bureau for Public Health, Charleston, West Virginia; 59 Kilte Awlaelo Health and Demographic Surveillance System, Ethiopia; 60 Arabian Gulf University, Manama, Bahrain; 61 Hamdan Bin Mohammed Smart University, Dubai, United Arab Emirates; 62 Department of Medical Mycology and Parasitology, School of Medicine, Mazandaran University of Medical Sciences, Sari, Iran; 63 Department of Epidemiology, Salah Azaiz Institute, Tunis, Tunisia; 64 Qatar University, Doha, Qatar; 65 Department of Public Health, School of Public Health, Shahid Beheshti University of Medical Sciences, Tehran, Iran; 66 University of Aberdeen, Aberdeen, United Kingdom; 67 Addis Ababa University, Debre Zeit, Ethiopia; 68 Wageningen University, Wageningen, Netherlands; 69 Department of Ophthalmology, Medical Faculty Mannheim, Ruprecht-Karls-University Heidelberg, Mannheim, Germany; 70 Hematology-Oncology and Stem Cell Transplantation Research Center, Tehran University of Medical Sciences, Tehran, Iran; 71 Jordan University of Science and Technology, Irbid, Jordan; 72 Health Services Academy, Islamabad, Pakistan; 73 Department of Microbiology and Immunology, College of Medicine and Health Sciences, United Arab Emirates University, Al Ain, United Arab Emirates; 74 Executive Board of the Health Ministers' Council for Cooperation Council States, Riyadh, Saudi Arabia; 75 Centre for Research and Action in Public Health, Faculty of Health, University of Canberra, Canberra, Australia; 76 University of British Columbia, Vancouver, Canada; 77 Research and Development Unit, Parc Sanitari Sant Joan de Deu (CIBERSAM), Barcelona, Spain; 78 Australian National University, Canberra, Australia; 79 Department of Zoology, Lahore College for Women University, Lahore, Pakistan; 80 Aintree University Hospital, National Health Service Foundation Trust, Liverpool, United Kingdom; 81 School of Medicine, University of Liverpool, Liverpool, United Kingdom; 82 Mansoura Faculty of Medicine, Mansoura, Egypt; 83 Imperial College London, London, United Kingdom; 84 Digestive Disease Research Institute, Tehran University of Medical Sciences, Tehran, Iran; 85 Howard University College of Medicine, Washington, District of Columbia; 86 University of Gondar, Gondar, Ethiopia; 87 School of Public Health, Mekelle University, Mekelle, Ethiopia; 88 School of Medicine, University of Adelaide, Adelaide, Australia; 89 Saudi Ministry of Health, Riyadh, Saudi Arabia; 90 College of Medicine, Alfaisal University, Riyadh, Saudi Arabia; 91 United Nations Population Fund, Lima, Peru; 92 Federal Ministry of Health, Khartoum, Sudan; 93 University of Pittsburgh Graduate School of Public Health, Pittsburgh, Pennsylvania; 94 Stellenbosch University, Cape Town, Western Cape, South Africa; 95 Institute for Global Health Innovations, Duy Tan University, Da Nang, Vietnam; 96 Aga Khan University, Karachi, Pakistan; 97 National Heart, Lung, and Blood Institute, Bethesda, Maryland; 98 University of Virginia, Charlottesville, Virginia; 99 Department of Community Medicine, School of Medicine, Alborz University of Medical Sciences, Karaj, Iran; 100 Contech International Health Consultants, Lahore, Pakistan; 101 Contech School of Public Health, Lahore, Pakistan; 102 Sina Trauma and Surgery Research Center, Tehran University of Medical Sciences, Tehran, Iran; 103 Hamad Medical Corporation, Doha, Qatar; 104 Society for Health and Demographic Surveillance, Suri, India; 105 Wonju College of Medicine, Yonsei University, Wonju, South Korea; 106 Institute for Poverty Alleviation and International Development, Yonsei University, Wonju, South Korea; 107 Department of Surgery, School of Medicine, Boston University, Boston, Massachusetts; 108 Suez Canal University, Ismailia, Egypt; 109 Naval Medical Research Center, Silver Spring, Maryland; 110 Golestan Research Center of Gastroenterology and Hepatology, Golestan University of Medical Sciences, Gorgan, Iran; 111 Muhimbili University of Health and Allied Sciences, Dar es Salaam, Tanzania; 112 Development Research and Projects Center, Abuja, Nigeria; 113 Department of Surgery and Comprehensive Cancer Center, Joan C. Edwards School of Medicine, Marshall University, Huntington, West Virginia; 114 Case Western Reserve University, Cleveland, Ohio; 115 Marshall University, Huntington, West Virginia; 116 Bahir Dar University, Bahir Dar, Ethiopia; 117 Faculty of Health Sciences, Hatter Institute for Cardiovascular Research in Africa, University of Cape Town, Cape Town, South Africa; 118 Department of Community Medicine, International Medical University, Kuala Lumpur, Malaysia; 119 Departments of Criminology, Law and Society, Sociology, and Public Health, University of California-Irvine, Irvine, California; 120 Westchester Medical Center, Valhalla, New York; 121 James Cook University, Cairns, Australia; 122 Department of Anesthesiology, University of Virginia, Charlottesville, Virginia; 123 Outcomes Research Consortium, Cleveland Clinic, Cleveland, Ohio; 124 Department of Anesthesiology, King Fahad Medical City, Riyadh, Saudi Arabia; 125 Department of Internal Medicine, Federal Teaching Hospital, Abakaliki, Nigeria; 126 Warwick Medical School, University of Warwick, Coventry, United Kingdom; 127 Federal Institute for Population Research, Wiesbaden, Germany; 128 German National Cohort Consortium, Heidelberg, Germany; 129 Addis Continental Institute of Public Health, Addis Ababa, Ethiopia; 130 Department of Biostatistics, School of Public Health, Kyoto University, Kyoto, Japan; 131 Jackson State University, Jackson, Mississippi; 132 University Hospital, Setif, Algeria; 133 Faculty of Medicine, Mansoura University, Mansoura, Egypt; 134 Melbourne School of Population and Global Health, University of Melbourne, Melbourne, Australia

## Abstract

Diarrheal diseases (DD) are leading causes of disease burden, death, and disability, especially in children in low-income settings. DD can also impact a child's potential livelihood through stunted physical growth, cognitive impairment, and other sequelae. As part of the Global Burden of Disease Study, we estimated DD burden, and the burden attributable to specific risk factors and particular etiologies, in the Eastern Mediterranean Region (EMR) between 1990 and 2013. For both sexes and all ages, we calculated disability-adjusted life years (DALYs), which are the sum of years of life lost and years lived with disability. We estimate that over 125,000 deaths (3.6% of total deaths) were due to DD in the EMR in 2013, with a greater burden of DD in low- and middle-income countries. Diarrhea deaths per 100,000 children under 5 years of age ranged from one (95% uncertainty interval [UI] = 0–1) in Bahrain and Oman to 471 (95% UI = 245–763) in Somalia. The pattern for diarrhea DALYs among those under 5 years of age closely followed that for diarrheal deaths. DALYs per 100,000 ranged from 739 (95% UI = 520–989) in Syria to 40,869 (95% UI = 21,540–65,823) in Somalia. Our results highlighted a highly inequitable burden of DD in EMR, mainly driven by the lack of access to proper resources such as water and sanitation. Our findings will guide preventive and treatment interventions which are based on evidence and which follow the ultimate goal of reducing the DD burden.

## Introduction

Diarrheal diseases (DD) are a major cause of childhood mortality globally, resulting in approximately 550,000 deaths among children under 5 years of age each year.^1^ Although mortality due to DD has declined significantly in children over the past 20 years, the incidence of childhood diarrhea in low-income countries (LICs) has not fallen appreciably.^1^ For those who survive these illnesses, repeated infections by enteric pathogens in the early years of life can lead to serious, lifelong health consequences such as environmental enteric dysfunction, growth faltering, impaired cognitive development, reduced immune response to infection and vaccinations, and death.^2^

Although a wide spectrum of bacterial, viral, and parasitic pathogens are responsible for infectious diarrhea worldwide, their etiologic contribution may vary depending on the geographic location of the study, study durations, or the population sampled.^3^ In addition, variations in the reported frequency of diarrhea may reflect the diagnostic tools used rather than the actual incidence of each pathogen.^4^ Furthermore, coinfections are common and more than one pathogen may be implicated in cases of diarrhea.

The Eastern Mediterranean Region (EMR) is home to more than 500 million people, representing a diverse group of 22 countries, including Arab states in north Africa, Gulf nations, and countries in west Asia. EMR countries have diverse historical backgrounds, political and social contexts, and fiscal and cultural influences on their health-care systems. The region has wide variation in per capita gross national product, ranging from a high of $134,420 in Qatar to a low of $2,000 in Afghanistan.^5^ Such wide variation has a major influence on overall health spending and results in substantial health inequities both within and across countries. Furthermore, the region has witnessed long years of political instability and conflicts, including the recent Arab Spring uprising and conflicts in countries such as Syria and Yemen.

Many countries in the EMR achieved important successes in the 1970s and 1980s with the support of the United Nations International Children's Emergency Fund and the World Health Organization (WHO) through the National Control of Diarrheal Diseases Project.^6^,^7^ For example, Egypt's program, which spanned from 1981 to 1991, was credited with significantly improving diarrheal case management.^8^–^10^ However, over the last two decades, the momentum has slowed.^11^ As of January 2016, rotavirus vaccines have been introduced through national immunization programs in only three (Djibouti, Republic of Sudan, and Yemen) of 11 Gavi-eligible countries in the region.^12^ The perceived lack of urgency relative to the major political and economic challenges facing the region has contributed to the current low level of awareness regarding the remaining burden of diarrhea. To date, there remains a lack of adequate research to address these diseases in many EMR countries.

Our objective in this report is to establish the size of the burden, distribution of pathogens, and risk factors for diarrhea in children and adults in the EMR for 1990 through 2013.

## Materials and Methods

### Study region.

The EMR countries were grouped according to per capita gross national income (GNI) into LICs (Islamic Republic of Afghanistan [Afghanistan], Djibouti, Somalia, Republic of Yemen [Yemen]); middle-income countries (MICs) (Arab Republic of Egypt [Egypt], Islamic Republic of Iran [Iran], Iraq, Jordan, Lebanon, Libya, Morocco, Pakistan, Palestine, Sudan, Syrian Arab Republic [Syria], Tunisia); and high-income countries (HICs) (Bahrain, Kuwait, Oman, Qatar, Saudi Arabia, and the United Arab Emirates [UAE]). We defined LICs as those having a per capita GNI of $1,045 or less, MICs as those with a per capita GNI between $1,046 and $12,735, and HICs as countries with per capita GNI of $12,736 or greater.

### Global burden of disease.

The Global Burden of Disease Study 2013 (GBD 2013) is a systematic, comprehensive effort to quantify health loss from 306 causes of diseases and injuries, 240 causes of death, and 79 risk factors by sex and age groups between 1990 and 2013 for 188 countries. GBD 2013's estimation process, including that for DD, has been described in detail elsewhere.^1^ The burden of DD was measured in disability-adjusted life years (DALYs), the sum of years of life lost due to premature death (YLLs) and years lived with disability (YLDs). The methods used to calculate each of these are summarized below.

### Mortality.

Cause-specific mortality estimates, including those for DD, were modeled using a Bayesian ensemble modeling process.^13^ Diarrhea mortality data included vital registration and verbal autopsy sources. The modeling process estimated the mortality rate due to diarrhea for both genders from 1990 to 2013 for all age groups in every country, and in subnational areas of some countries. Covariates, such as improved water and sanitation sources, malnutrition, and population density were considered. The ensemble model approach allowed for a suite of models, weighted by out-of-sample predictive validity, to inform the final estimates.

### Morbidity.

As with mortality, morbidity is modeled at every year, gender, age, and geographic location in the GBD study. We used DisMod-MR 2.0, a Bayesian meta-regression mixed effects model, to calculate morbidity. DisMod-MR was originally developed for GBD 2010 to address statistical challenges in estimating nonfatal health outcomes and synthesizing sparse and heterogeneous epidemiological data. It uses nonlinear mixed effects models that include country- and study-level covariates to generate priors for increasingly detailed geographic regions in an analytic cascade. Prevalence, incidence, recovery, and death are related in a series of ordinary differential equations within DisMod-MR. Basically, it is a mathematical modeling technique that takes data collected from different sources, corrects for inconsistencies, and fills in gaps when data are incomplete, ultimately producing estimates of disease burden by age, sex, and country. A full description of DisMod-MR is available elsewhere.^14^–^16^ Diarrhea prevalence and incidence data from a systematic literature review and population-representative surveys informed the meta-analytic model and estimates.

### Etiologies.

Diarrhea cases and deaths were attributed to pathogens using a counterfactual approach. A systematic literature review on the proportion of diarrhea cases that test positive for a set of pathogens is used in the DisMod-MR framework to estimate the age, sex, year, and geographic distribution of diarrheal pathogens. The population-attributable fraction (PAF) is used to parse the fraction of diarrhea cases and deaths due to each pathogen. The PAF is calculated as

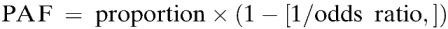


Where “proportion” is the proportion of cases positive for a pathogen and “odds ratio” (OR) is the odds of diarrhea given pathogen detection. The ORs were derived from the Global Enteric Multicenter Study (GEMS), a multisite case-control study of moderate-to-severe diarrhea in children under 5 years of age,^17^ using a mixed effects conditional logistic regression model. With the exception of Pakistan, where the ORs were calculated from a model including only the Pakistan GEMS site, the ORs were calculated from a model that included all GEMS sites.

*Vibrio cholerae* and *Clostridium difficile* were estimated separately from the other pathogens in GBD. Cholera cases were estimated using data from previous studies compared with WHO case notification data to estimate underreporting. Cholera deaths were estimated using case fatality data in DisMod-MR. Since *C. difficile* is frequently associated with hospital and health-care utilization, hospital incidence data were modeled in DisMod-MR 2.0.

### Risk factors.

We also assessed diarrheal DALYs, YLLs, and YLDs attributable to childhood stunting (below −2 standard deviations of the median height of a reference population), suboptimal breastfeeding (nonexclusive breastfeeding and discontinued breastfeeding), vitamin A deficiency, zinc deficiency, and water, sanitation, and hygiene (WASH).

### Data analysis.

The average decrease in deaths, DALYs, YLLs, and YLDs was calculated by subtracting the mean estimate in 1990 from the mean estimate in 2013, divided by 23 years. All reported rates are per 100,000 persons and are not age standardized. Uncertainty for deaths, DALYs, and attributable fractions in GBD were derived from 1,000 draws of the variables used to estimate these outcomes. For example, the ORs, pathogen prevalence among diarrhea cases, and mortality rates are from normal or log-normal distributions and were combined at the draw level to estimate pathogen-specific diarrhea mortality. The 95% uncertainty intervals (UIs) then are the 2.5 and 97.5 percentiles of these estimates. This analysis was conducted using Stata/SE 13.1 (StataCorp LP, College Station, TX) and maps were generated using the ggplot2^18^ package in R 3.2.2 (R Foundation for Statistical Computing, Vienna, Austria).

## Results

### Deaths.

There were 128,024 (95% UI = 88,729–172,361) diarrhea-related deaths in the EMR in 2013, with an overall rate of 21 diarrheal deaths per 100,000 persons (95% UI = 14–28) (Table 1). The majority of these deaths occurred among children under 5 years of age (68.9%) (88,172, 95% UI = 52,794–129,711) and adults over 70 years of age (11.9%) (15,278, 95% UI = 9,491–22,518). There were no consistent differences in diarrhea deaths per 100,000 between males and females (Supplemental Figure 1). There were some exceptions to the general pattern of children under 5 years of age bearing the greatest burden. In Djibouti, in the year 2000, diarrhea deaths per 100,000 were greater among those over 70 years of age (680, 95% UI = 394–1,088) than among those under 5 years of age (187, 95% UI = 84–332). In neighboring Somalia, death rates were also higher for individuals over 70 years of age versus individuals under 5 years of age at each time point.

**Figure 1.**
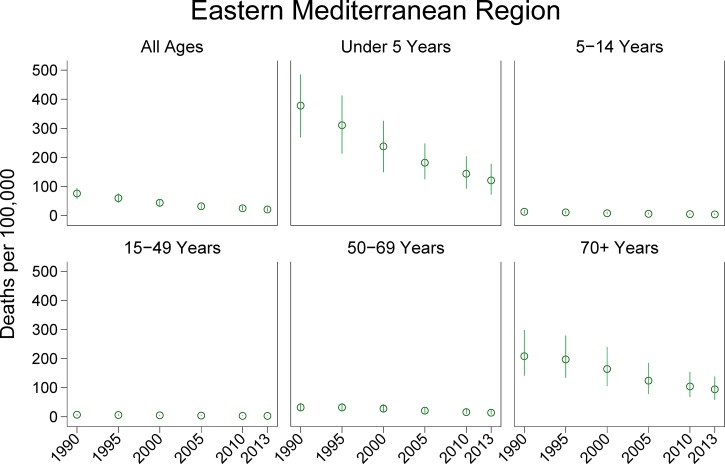
Diarrheal disease-associated deaths in the Eastern Mediterranean Region, 1990–2013.

**Figure 2.**
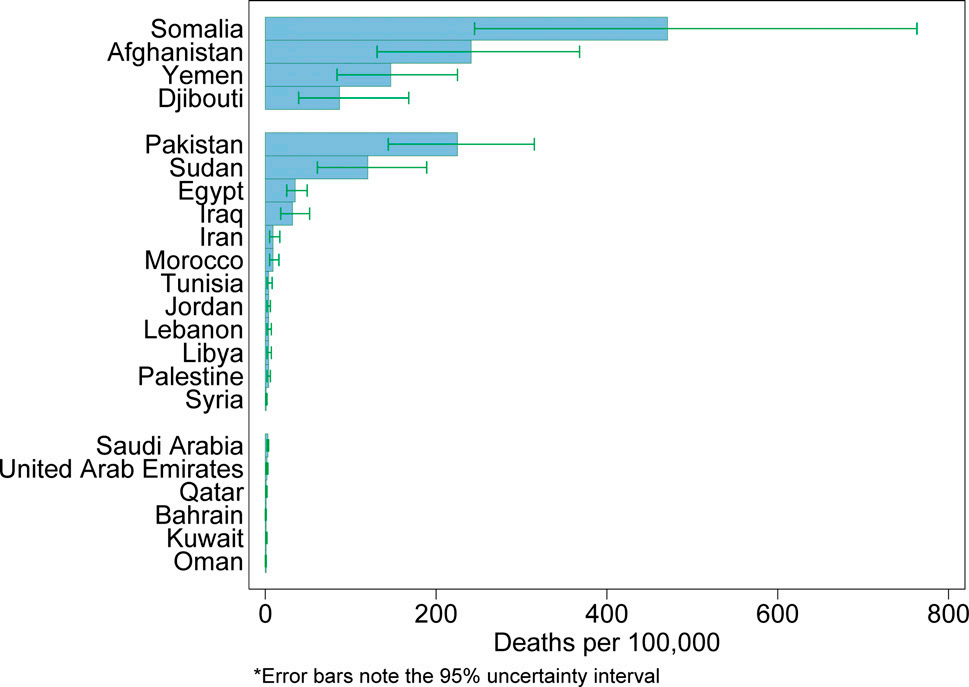
Diarrheal disease-associated death rates among children under 5 years of age in the Eastern Mediterranean Region, sorted by low, middle, and high income per capita gross national income, 2013.

Graphs of diarrhea deaths per 100,000 demonstrated a steady decrease from 1990 to 2013, with the most notable declines among children under 5 years of age and individuals ≥ 70 years of age (Figure 1

). For all ages, diarrhea death rates decreased by an average of 2.39% deaths per 100,000 per year during this period. For those under 5, 5–14, 15–49, 50–69, and over 70 years of age, the average decrease in the diarrheal death rate was 11.17%, 0.39%, 0.17%, 0.78%, and 4.96% per 100,000 per year, respectively. The 2013 diarrhea-associated death rates were stratified by country and per capita GNI (Figure 2

). Diarrhea deaths per 100,000 children under 5 years of age ranged from one (95% UI = 0–1), in Bahrain and Oman (HICs), to 471 (95% UI = 245–763), in Somalia (a LIC) (Supplemental Table 1). Among MICs, Pakistan (225, 95% UI = 144–315) and Sudan (120, 95% UI = 61–189) had diarrhea death rates in excess of their economic peers. Overall, diarrhea mortality appeared to be related to per capita GNI rather than geographical location.

### Years of life lost due to premature mortality.

Nearly 9 million (8,935,214, 95% UI = 5,865,116–12,571,874) YLLs were attributable to diarrhea in the EMR in 2013. Children under 5 years of age bore the majority (84.5%) of this burden (7,553,654, 95% UI = 4,517,051–11,112,683), and had a rate of 10,361 (95% UI = 6,196–15,242) YLLs per 100,000. Children under 5 years of age in Bahrain (57, 95% UI = 34–88), Oman (59, 95% UI = 31–108) and Qatar (95, 95% UI = 53–167) experienced the lowest rates of YLLs per 100,000 in the region. On the other hand, children in Somalia (40,238, 95% UI = 20,975–65,112) and Afghanistan (20,675, 95% UI = 11,160–31,515), two countries with the longest history of ongoing civil wars in the region, experienced the highest rates.

### Years lived with disability.

Diarrhea was responsible for a total of 1,002,096 (95% UI = 673,662–1,392,552) YLDs in 2013 in the region, with children under 5 years of age contributing more than half of this burden (586,935, 95% UI = 391,944–820,782). The rate of YLDs per 100,000 among children under 5 years of age was 805 (95% UI = 538–1,126) and ranged from 177 (95% UI = 106–263) in Djibouti to 974 (95% UI = 662–1,357) in Afghanistan.

### Disability-adjusted life years.

Nearly 10 million DALYs were attributable to diarrhea (9,937,310, 95% UI = 6,802,456–13,650,080), with an overall rate of 1,610 (95% UI = 1,102–2,212) DALYs per 100,000 in the EMR in 2013 (Table 2). The majority of DALYs were borne by those under 5 years of age (81.9%) (8,140,589, 95% UI = 5,082,597–11,726,801) and those 15–49 years of age (7.4%) (737,925, 95% UI = 498,093–1,058,052). There were no consistent differences in diarrhea-associated DALYs between males and females.

The average decrease in the number of DALYs over this time period in the region as a whole was 568,035 per year, with the majority of this decrease among those under 5 and 5–14 years of age (543,789 and 23,734 DALYs per year, respectively). The pattern for diarrhea DALYs among those under 5 years of age closely followed that for diarrheal deaths. DALY rates ranged from 739 (95% UI = 520–989) in Syria to 40,869 (95% UI = 21,540–65,823) in Somalia.

In each country, diarrhea DALYs decreased over time and children under 5 years of age consistently contributed the majority of diarrheal DALYs (Supplemental Figure 2).

### Etiologies.

**Figure 3.**
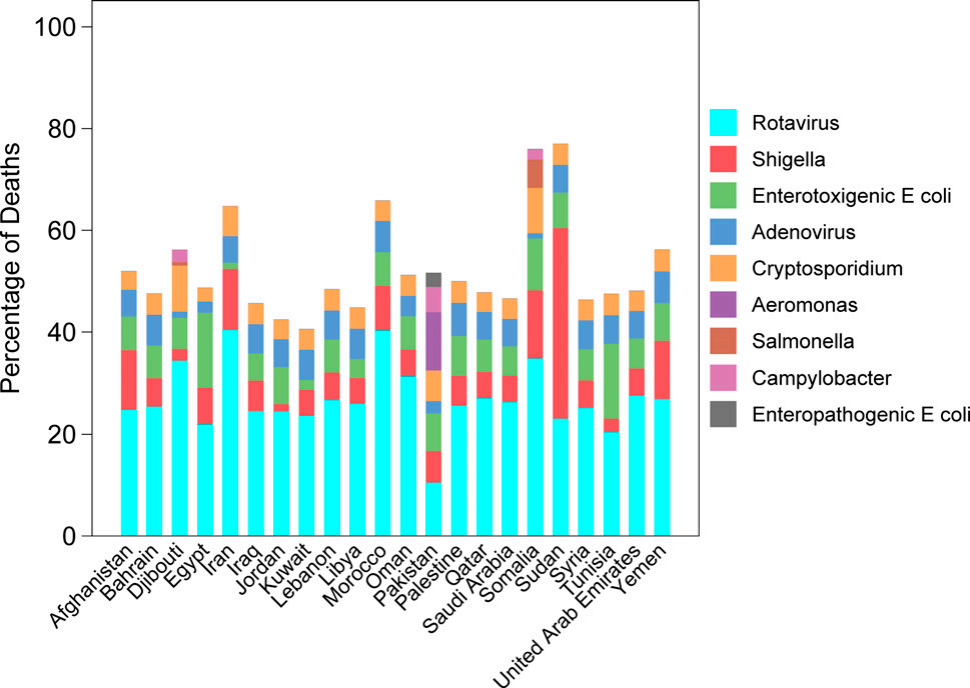
The proportion of diarrheal deaths due to known pathogens,* among children under 5 years of age in the Eastern Mediterranean Region, 2013 (*excluding cholera and *Clostridium difficile*).

The proportion of deaths attributable to specific etiologies in 2013, excluding cholera and *C. difficile*, are presented in Figure 3

. Generally, rotavirus infection was the largest contributor to the diarrheal burden of disease. However, *Shigella* was the greatest contributor to diarrheal deaths and DALYs in Sudan. When also considering cholera and *C. difficile*, the total proportion of attributable deaths was greater than 100% (more than one attributable pathogen per diarrheal episode) for Jordan, Palestine, Syria, Bahrain, and Oman (Supplemental Figure 3). These same five countries also had a relatively large proportion of diarrheal deaths attributed to cholera in 2013 and an increasing cholera burden in the recent years (Supplemental Figure 4). Graphs of the relative proportions of known etiologies leading to diarrheal DALYs for children under 5 years of age are provided in Supplemental Figure 5.

### Risk factors.

In 2013, the two risk factors that contributed to the greatest number of diarrhea-attributable deaths (Supplemental Figure 6) and DALYs (Supplemental Figure 7) among those under 5 years of age were WASH and suboptimal breastfeeding. Rates of suboptimal breastfeeding–related deaths ranged from zero (0, 95% UI = 0–1) in Bahrain and Oman to 232 (95% UI = 105–405) in Somalia. The same pattern was seen with rates of WASH-related deaths, where Bahrain and Oman had a rate of one (95% UI = 0–1) and Somalia's rate was 457 (95% UI = 238–734). WASH-related deaths and DALYs were somewhat greater in LICs, and the burden due to suboptimal breastfeeding was generally greater in HICs (Supplemental Figures 8 and 9).

## Discussion

We reported the most comprehensive assessment of DD burden and the contributions of specific pathogens and risk factors in the EMR to date. In 2013, the estimated diarrhea-associated deaths and DALYs were more than 125,000 and nearly 10 million, respectively. We also found substantial variation within the region, with LICs and MICs experiencing social unrest bearing the vast majority of diarrheal burden.

Our estimates are comparable with others from recent publications. A systematic literature review published in 2008 estimated diarrhea deaths among those under 5 years of age in 13 EMR countries (Bahrain, Cyprus, Iran, Jordan, Kuwait, Lebanon, Libya, Oman, Qatar, Saudi Arabia, Syria, Tunisia, and the UAE) to be 12,000 (UI = 10,000–14,000), and in nine other EMR countries (Afghanistan, Djibouti, Egypt, Iraq, Morocco, Pakistan, Somalia, Sudan, and Yemen) to be 221,000 (UI = 190,000–250,000).^19^ Our estimated number of DD-associated deaths in this age group, which combines these two subregions, was similar. In addition, although we did not follow the same grouping of countries in our study, the countries for which we estimated the highest diarrhea burden were all included in the “high-mortality” countries according to the study. In addition, a 2013 publication estimated the number of DD-associated deaths in the EMR among children under 5 years of age in 2011 to be 96,000 (UI = 64,200–153,300).^20^ This is comparable to our finding of 88,172 (UI = 52,794–129,711) and suggests a slight increase in the number of deaths.

Our data clearly illustrated the gross health inequity in the region, with HICs experiencing a nominal diarrhea burden compared with the substantial burden in all LICs and some MICs. However, what cannot be observed in our data are the potential inequities in access to infrastructure and services within individual countries of the EMR. Not only do wealthier and urban communities have better access to proper infrastructure for water and sanitation, they also tend to have the education and financial resources to properly use point-of-use water treatment modalities. Furthermore, the introduction of vaccines against diarrheal pathogens may exacerbate inequalities in diarrhea burden. For example, although rotavirus infection was the largest contributor to the diarrheal burden of disease, in some countries in the region, rotavirus vaccine is only available in the private market. This means that wealthier families, who have less need for the vaccine, will gain the primary benefit from its availability. This is troubling because economic analyses of rotavirus vaccine introduction among a number of EMR countries have uniformly suggested that vaccine introduction would be cost-beneficial from a societal perspective.^6^,^21^–^23^ One study in Somalia (the only LIC country) suggested that introduction of a rotavirus as special immunization program during a complex humanitarian emergency would meet WHO cost-effectiveness benchmarks.^24^

A unique contribution of this analysis is the inclusion of all age groups. Due to the high disease burden in young children, nearly all diarrhea interventions and most diarrhea burden studies are limited to those under 5 years of age.^19^,^20^,^25^ However, the burden among those over 70 years of age is substantial, with DD-associated deaths totaling nearly one-sixth of the number among those under 5 years of age. The elderly may face increased diarrhea risk due to immunosenescence and comorbidities, which may also necessitate special consideration in their treatment.^26^ The increasing nature of cholera burden in five EMR countries (Jordan, Palestine, Syria, Bahrain, and Oman) is a cause of concern, as neighboring countries remain at a high risk of transmission due to presence and movement of refugee populations among them.

We found no systematic difference in under-5 diarrhea deaths or DALYs when comparing females to males. We hypothesized that there may exist evidence of differential diarrhea mortality burden by sex. A previous analysis of global demographic and health survey (DHS) data reported that girls 1–4 years of age, particularly in the Middle Eastern crescent, are at a mortality disadvantage compared with boys,^27^ perhaps due to differences in health-care access and nutritional status. Furthermore, an Egyptian study found some evidence that, even when parents sought care for their daughters with diarrhea, regional health-care providers provided biased treatment in favor of boys.^28^ However, more recent publications, such as a 2009 verbal autopsy study in Iraq, found no difference in under-5 mortality by sex.^29^

Our study has several limitations and strengths. First, while our modeling process seeks to make use of all available data, the number of relevant publications in the region is limited and unbalanced between countries. However, our hierarchical modeling approach allows us to “borrow” strength across time and geography to generate the best possible estimates. Second, since we only account for the acute phase of diarrhea in our YLD estimates, the resulting DALYs severely underestimate diarrhea-associated morbidity. In GBD 2016, we expect to include long-term sequelae such as stunting and cognitive impairment,^30^,^31^ which will better estimate the true burden of disease. Third, the reported distribution of diarrhea etiologies was based on traditional laboratory detection techniques,^17^ which may be less sensitive and specific than molecular methods. For this reason, our future GBD estimates will incorporate a GEMS reanalysis based on standardized quantitative polymerase chain reaction data for all pathogens.^32^ Despite these limitations, this analysis also has several strengths. GBD methodology ensures internal consistency so that morbidity and mortality cannot be simultaneously ascribed to competing causes and allows for comparability between countries and across regions.

## Conclusions

Although there were substantial improvements in DD-related morbidity and mortality, the health inequities revealed in our data show that more action is needed to reduce the burden of diarrhea in the EMR, especially in lower-income countries and countries experiencing political and social unrest. A coordinated approach that involves prevention and treatment is needed to address the multiple causes of DD. Regional health systems need to be strengthened to achieve the widespread availability and use of oral rehydration salts, improved rates of breastfeeding, improved nutrition, better sanitation and hygiene, and increased coverage of measles immunization. Due to the high relevance of rotavirus infections in EMR,^33^ there is also an urgent need to roll out rotavirus vaccine in the region through government immunization programs that would ensure access for the children who are most in need. In addition, regional governments should begin deliberation to integrate *Shigella*, enterotoxigenic *Escherichia coli*, and other diarrheal vaccines that are currently in preclinical and clinical trials into their expanded programs on immunization as soon as they are approved and licensed.

## Supplementary Material

Supplemental Datas.

## Figures and Tables

**Table 1:** Diarrheal disease-associated deaths in the EMR, 2013

Age (years)	Sex	Deaths (UI)	Rate* (UI)	% of total†
All ages	Male	64,690 (38,893–95,066)	20 (12–30)	3.29
	Female	63,334 (38,706–92,897)	21 (13–31)	3.88
	Total	128,024 (88,729–172,361)	21 (14–28)	3.56
< 5	Male	44,346 (20,941–72,951)	118 (56–195)	11.19
	Female	43,826 (21,668–71,417)	124 (61–201)	12.70
	Total	88,172 (52,794–129,711)	121 (72–178)	11.90
5–14	Male	2,484 (1,113–4,690)	4 (2–7)	4.68
	Female	3,105 (1,467–5,590)	5 (2–9)	7.17
	Total	5,589 (3,175–9,011)	4 (2–7)	5.80
15–49	Male	4,634 (2,381–7,942)	3 (1–5)	1.14
	Female	4,878 (2,537–8,189)	3 (2–5)	1.81
	Total	9,512 (5,765–14,738)	3 (2–4)	1.41
50–69	Male	5,091 (2,584–8,884)	15 (8–27)	0.97
	Female	4,383 (2,105–7,859)	13 (6–24)	1.13
	Total	9,474 (5,487–15,012)	14 (8–23)	1.04
70+	Male	8,136 (4,253–13,493)	106 (55–175)	1.38
	Female	7,142 (3,800–12,096)	83 (44–140)	1.22
	Total	15,278 (9,491–22,518)	94 (58–138)	1.30

EMR = Eastern Mediterranean Region; UI = uncertainty interval.

* Rate is per 100,000.

† Percent of total deaths in the EMR for the specified age group and sex.

**Table 2:** Diarrheal disease-associated DALYs in the Eastern Mediterranean Region, 2013

Age (years)	Sex	DALYs (UI)	Rate* (UI)	% of total†
All ages	Male	5,003,063 (2,917,614–7,527,475)	1,575 (918–2,369)	4.51
	Female	4,934,247 (2,959,335–7,385,260)	1,648 (988–2,466)	5.14
	Total	9,937,310 (6,802,456–13,650,080)	1,610 (1,102–2,212)	4.80
< 5	Male	4,110,927 (2,083,037–6,565,118)	10,979 (5,563–17,533)	11.50
	Female	4,029,662 (2,105,785–6,409,279)	11,363 (5,938–18,073)	12.97
	Total	8,140,589 (5,082,597–11,726,801)	11,166 (6,971–16,085)	12.19
5–14	Male	267,327 (148,924–452,243)	400 (223–678)	3.33
	Female	306,681 (169,331–506,004)	486 (268–802)	4.45
	Total	574,008 (367,111–863,442)	442 (283–665)	3.84
15–49	Male	363,936 (223,641–561,754)	211 (130–326)	0.96
	Female	373,988 (224,349–577,646)	235 (141–363)	1.16
	Total	737,925 (498,093–1,058,052)	222 (150–319)	1.05
50–69	Male	158,484 (87,613–261,122)	476 (263–785)	0.78
	Female	134,679 (72,309–228,946)	407 (219–692)	0.80
	Total	293,163 (182,407–446,130)	442 (275–672)	0.79
70+	Male	102,390 (57,014–165,506)	1,330 (741–2,150)	1.15
	Female	89,236 (51,181–142,531)	1,035 (593–1,652)	1.01
	Total	191,626 (124,949–275,176)	1,174 (765–1,686)	1.08

DALY = disability-adjusted life years; EMR = Eastern Mediterranean Region; UI = uncertainty interval.

* Rate is per 100,000.

† Percent of total DALYs in the EMR for the specified age group and sex.
