# Supplementary material for: Burden of Diarrhea in the Eastern Mediterranean Region, 1990–2013: Findings from the Global Burden of Disease Study 2013
Source: Am J Trop Med Hyg. 2016 Dec 7;95(6):1319–29. doi: 10.4269/ajtmh.16-0339 (PMC5154365; doi:10.4269/ajtmh.16-0339)
Supplement: Supplementary file 1 [file SD2.pdf]

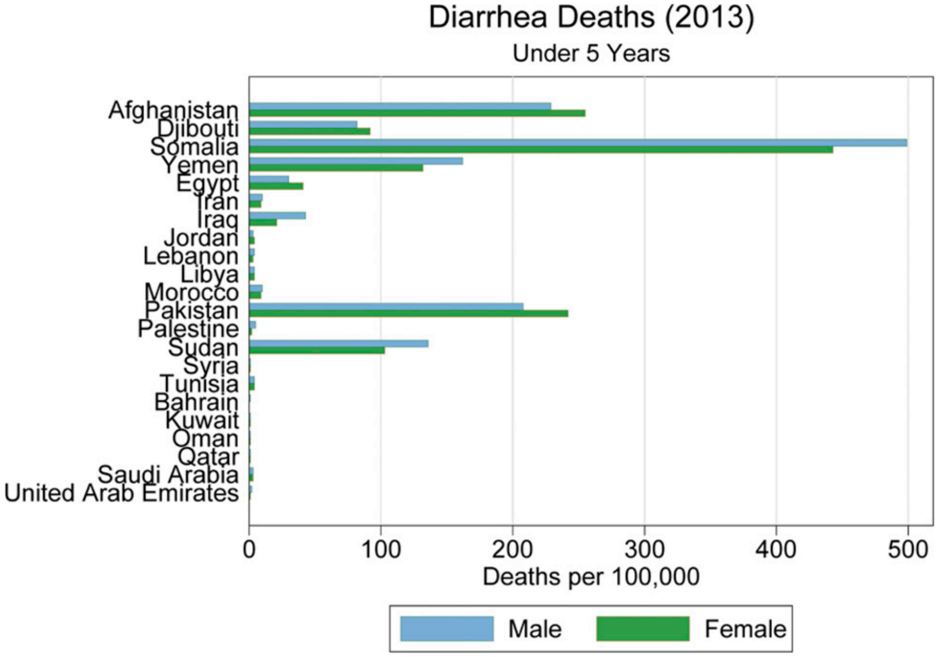


SUPPLEMENTAL FIGURE 1. Diarrheal disease-associated deaths in the Eastern Mediterranean Region by sex, 2013.

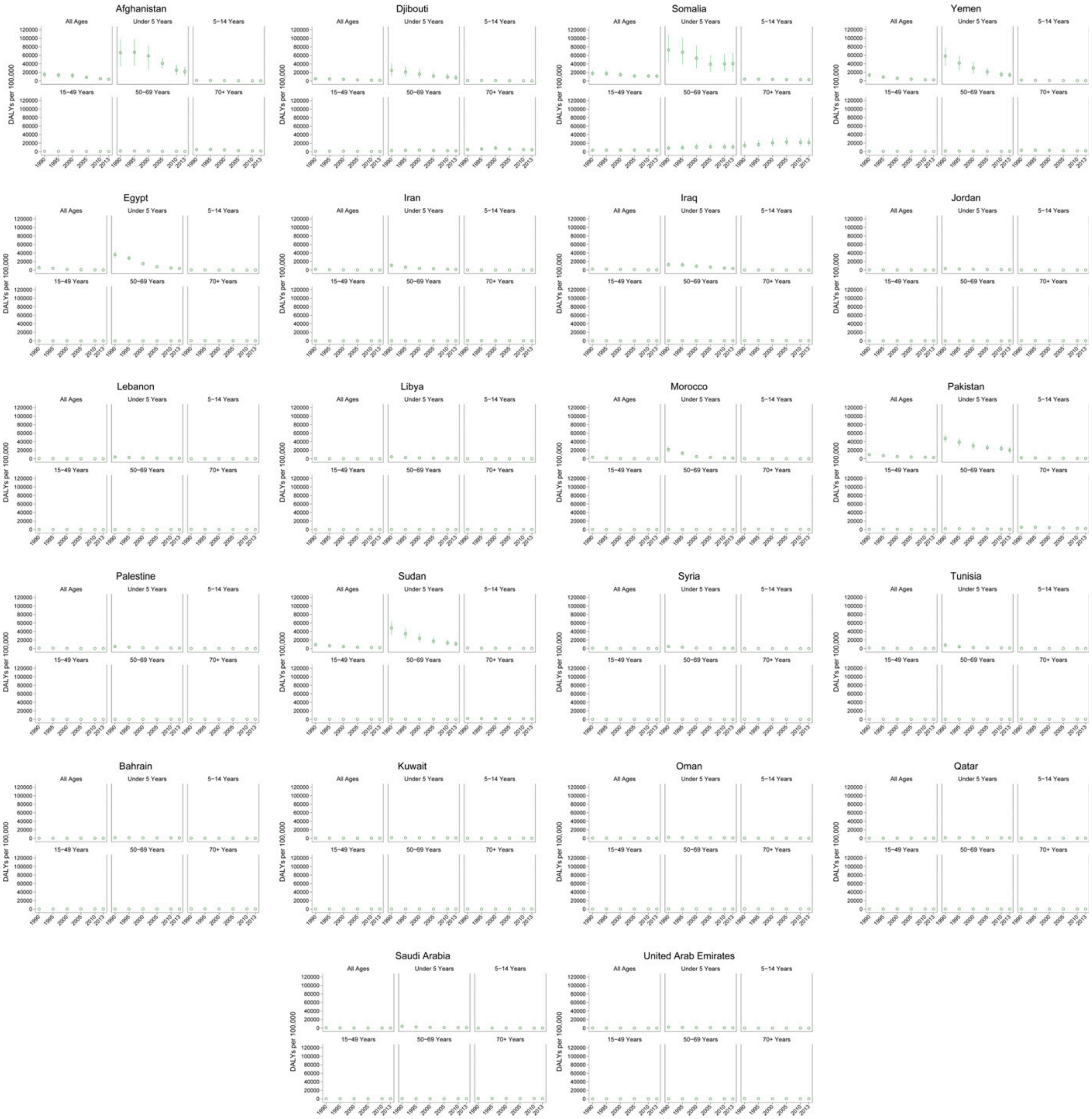


SUPPLEMENTAL FIGURE 2. Changes in diarrheal disease-associated disability-adjusted life years (DALYs) in the Eastern Mediterranean Region by country, 1990–2013.

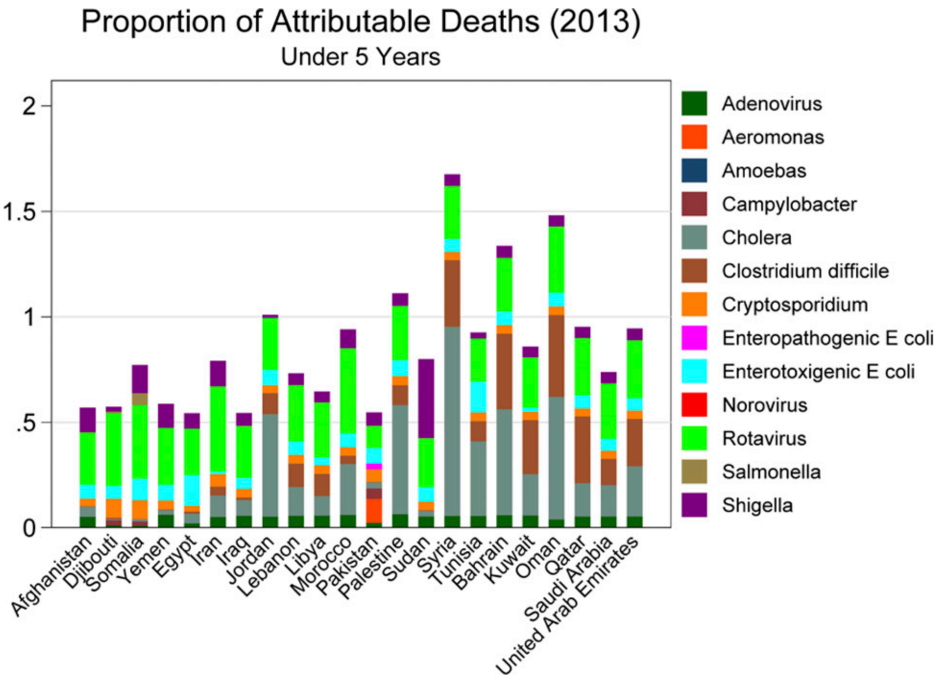


SUPPLEMENTAL FIGURE 3. The proportion of diarrheal disease-associated deaths among children under 5 years of age attributable to specific etiologies in the Eastern Mediterranean Region, 2013.

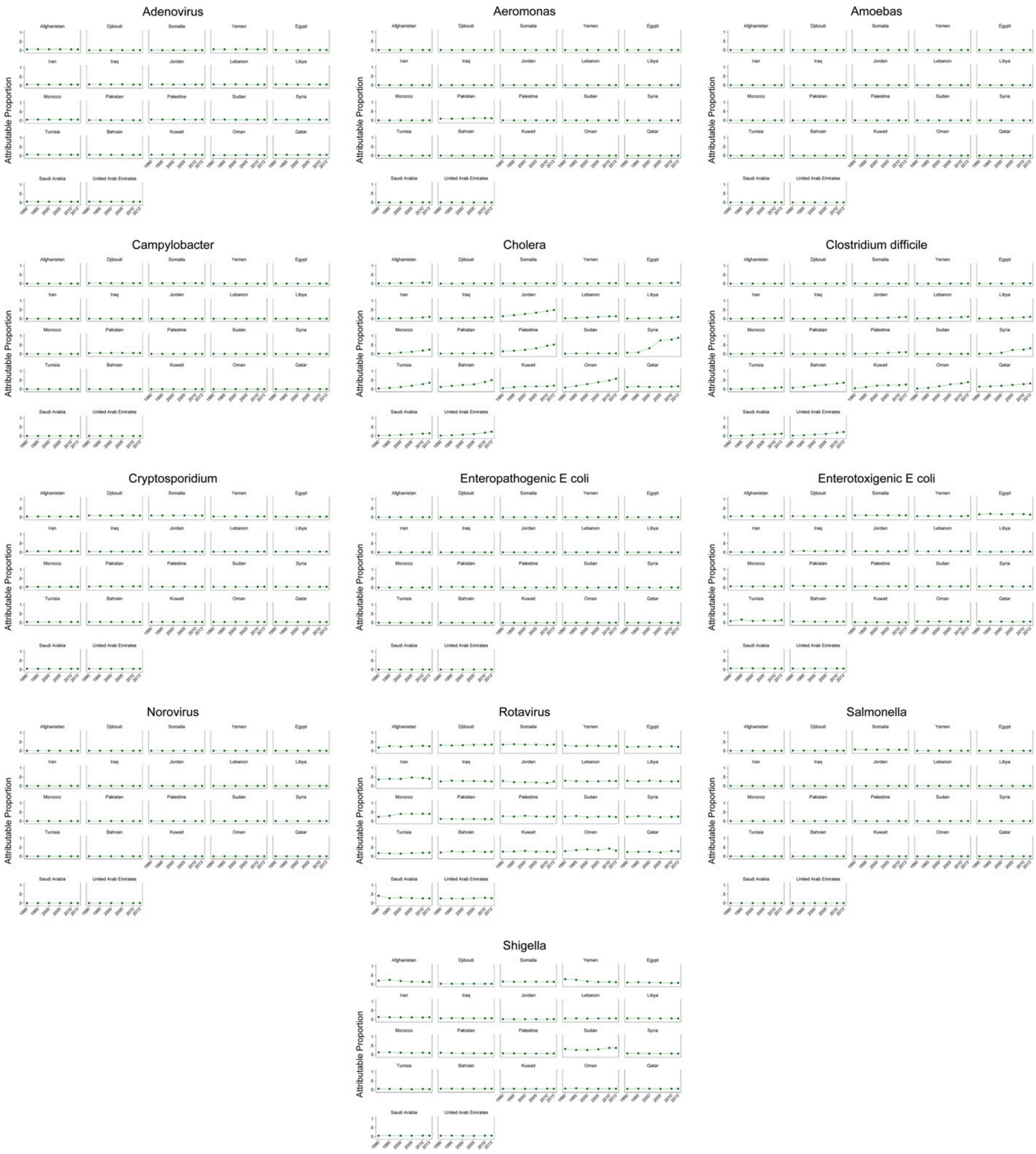


Supplemental Figure 4. The proportion of diarrheal disease-associated deaths attributable to specific etiologies in the Eastern Mediterranean Region, 1990–2013.

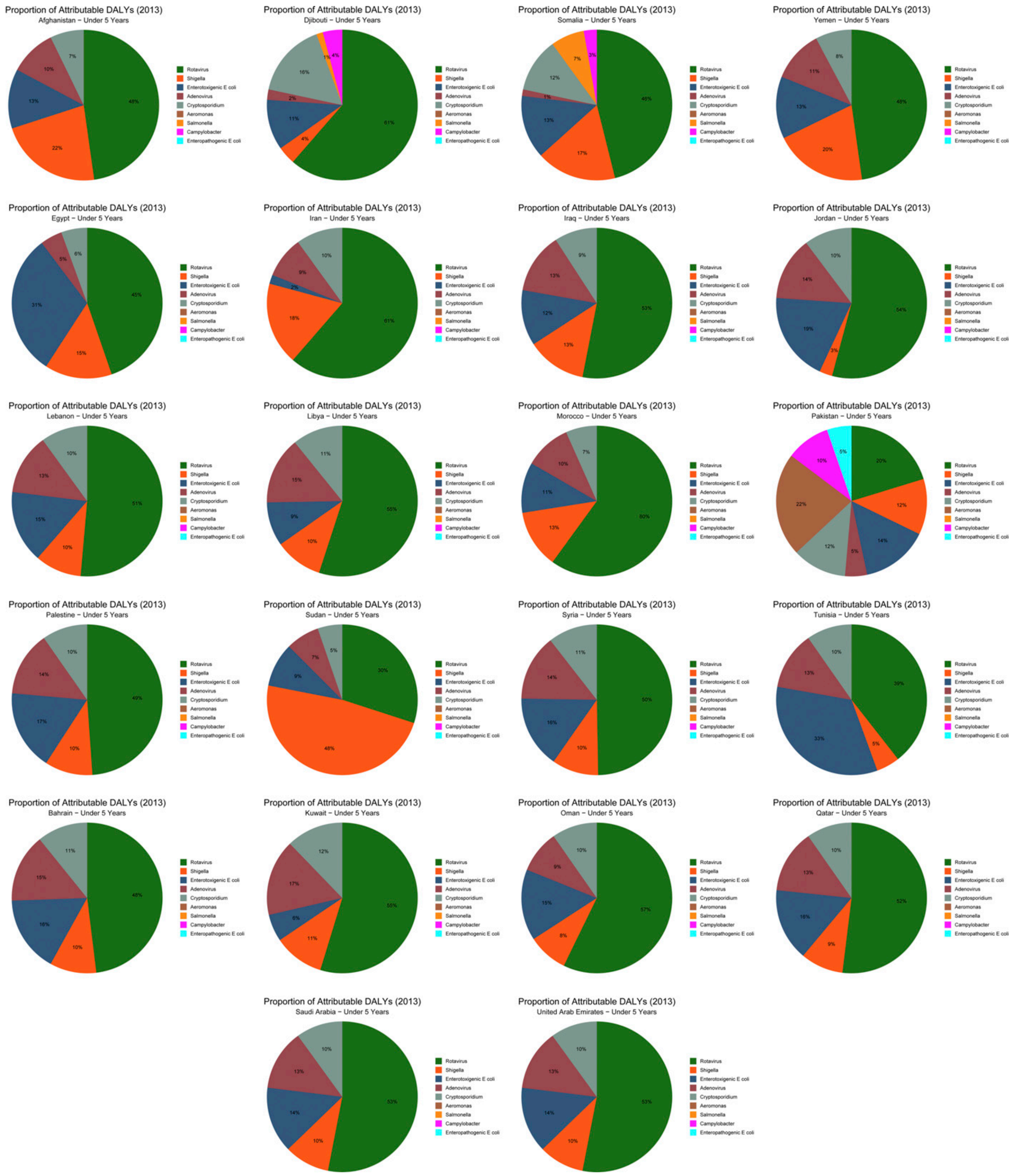


Supplemental Figure 5. The relative proportion of diarrheal disease-associated disability-adjusted life years (DALYs) attributable to specific etiologies among children under 5 years of age in the Eastern Mediterranean Region, by country in 2013.

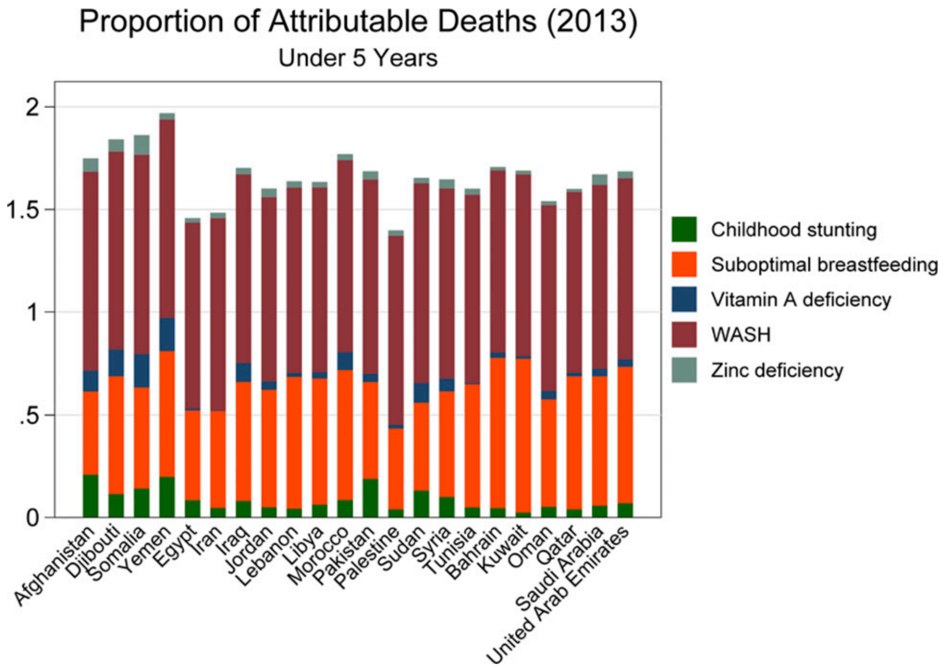


SUPPLEMENTAL FIGURE 6. The proportion of diarrheal disease-associated deaths among children under 5 years of age attributable to specific risk factors in the Eastern Mediterranean Region, 2013.

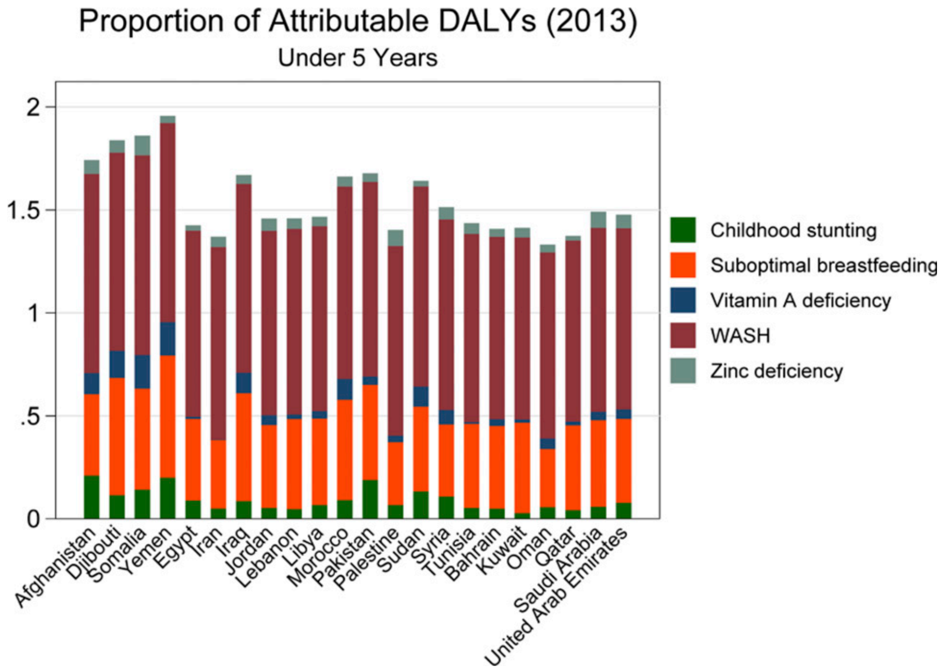


SUPPLEMENTAL FIGURE 7. The proportion of diarrheal disease-associated disability-adjusted life years (DALYs) among children under 5 years of age attributable to specific risk factors in the Eastern Mediterranean Region, 2013.

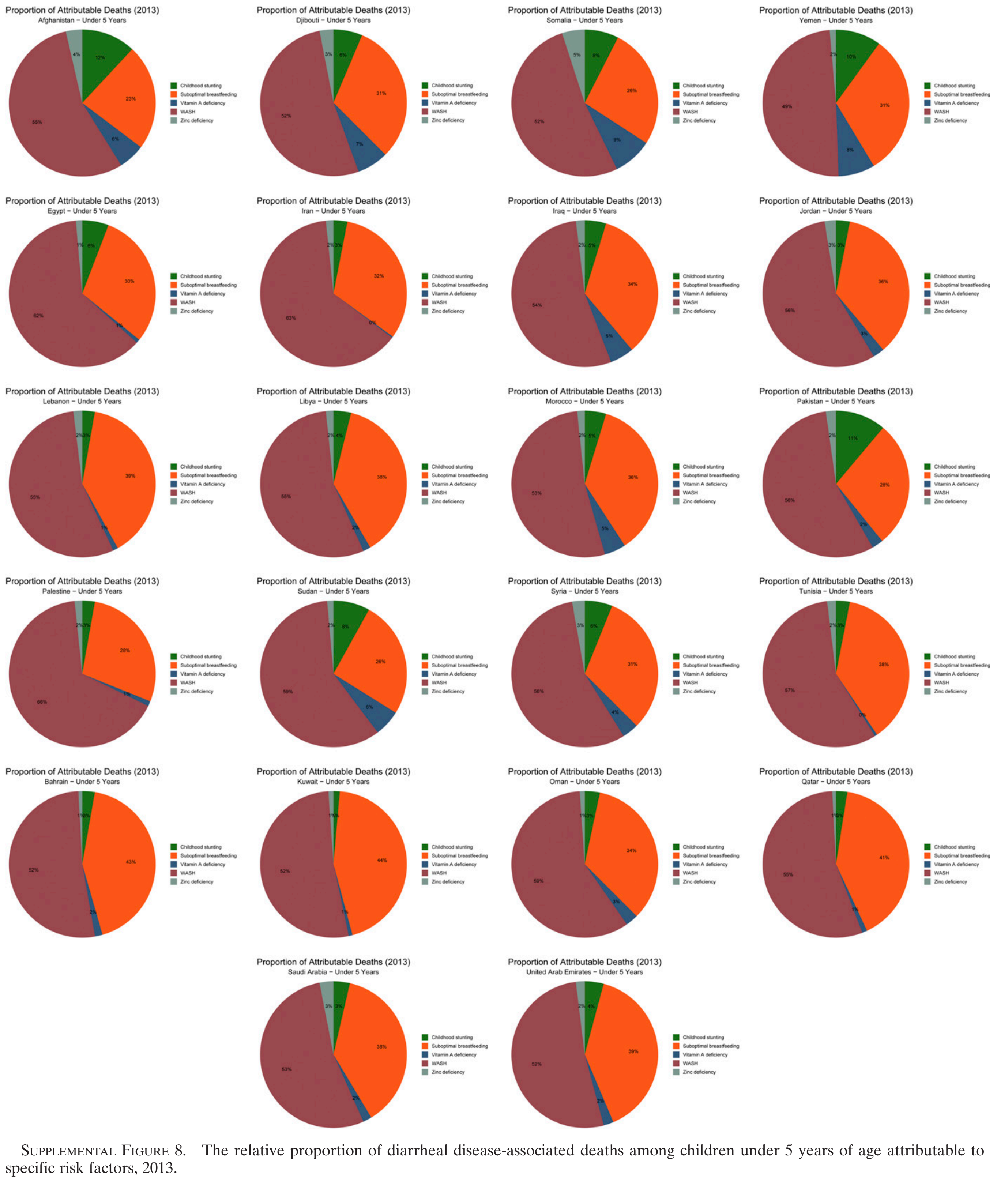


Supplemental Figure 8. The relative proportion of diarrheal disease-associated deaths among children under 5 years of age attributable to specific risk factors, 2013.

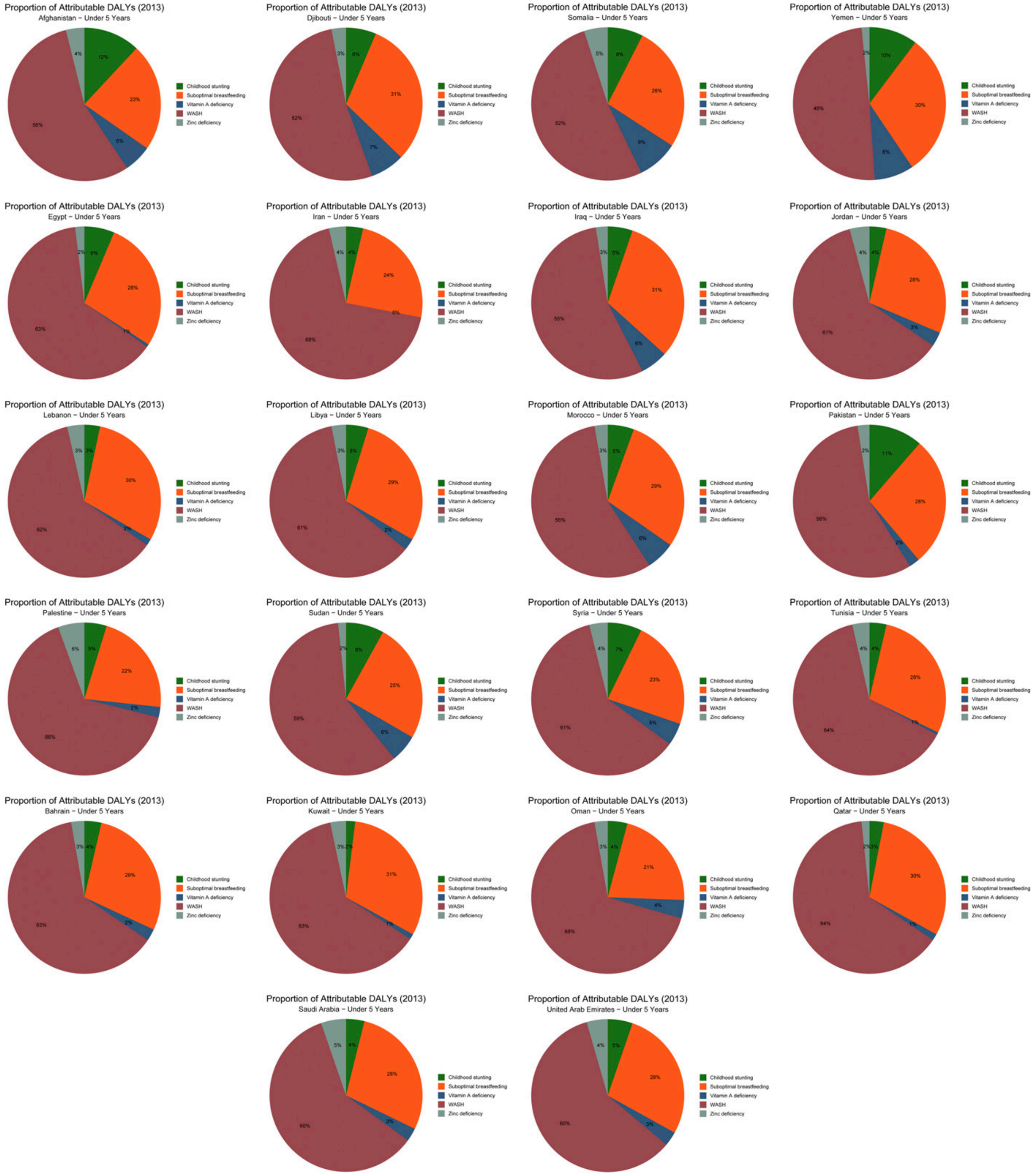


SUPPLEMENTAL FIGURE 9. The relative proportion of diarrheal disease-associated disability-adjusted life years (DALYs) among children under 5 years of age attributable to specific risk factors, 2013.
